# Lunatic Asylums in Ireland

**Published:** 1851-01-01

**Authors:** 


### Art. IV.—LUNATIC ASYLUMS IN IRELAND.

Having taken a rapid glance at the origin, progress, and present condition of our own lunatic asylums, in our last number, we turn, with no ordinary interest, to consider the subject so far as it relates to the sister kingdom.

Bad as have been the legislative provisions for England, matters do not seem to have worn a much better aspect in the sister kingdom. By reference to parliamentary papers, we find that no notice was taken, before the Act of Union, of the condition of the Irish lunatic. In fact, we might have supposed that lunatics had no existence in that country. Some provision, indeed, had been made in regard to the county of Cork; but it was too limited in its nature, and so confined in its operation, that it is almost unnecessary to mention it as an exception to the rest of Ireland. In 1804, a Committee of the House of Commons passed a series of resolutions to the effect that certain acts of parliament (27 Geo. III.), not having been fully carried out, inasmuch as that provisions had been made only in Dublin for 118 lunatics and idiots; in Cork, for 90; in Waterford, for 25; further and more decided legislation became necessary. In 1806, a kind of half-prison, half-medical act (known as 46 Geo. III. c. xcv.) was passed; its second clause empowered a sum, not exceeding 100*l.* per annum, to be levied for wards for the reception, in any hospital, &c., of a county, city, or county of a town, of idiots or lunatics. This Act had been found necessary on the grounds, as the committee stated, that, "with the exception of the four above-named towns, it did not appear that any institution maintained in any degree at the public expense, exists in any part of Ireland for the reception of such cases." This was indeed a sad picture of the state of the lunatic poor of Ireland, at a period (1805-6) when her population, by official returns, amounted to 5,395,000 souls. This Act was, moreover, passed in 1806, mainly on the ground "that the poor and the lunatic were confined in the same houses—a practice from which the most distressing inconveniences resulted, the method of confinement being only fitted for malefactors," &c.

The fearful struggle in which Great Britain was then and for many years subsequently engaged, rendered almost any minor subject distasteful, until she had to some extent freed her homes from the terrors of the continental emperor; and nothing appears to have been done until in 1810-11, the evil became so pressing and so enormous, that further postponement was impossible. As an experiment, the Richmond Asylum, in Dublin, was empowered to receive certain grants from the

British treasury; and, in 1815, accommodation was opened for 200 patients, or rather prisoners, for as yet we have not arrived at the point of separation. Even that accommodation appeared almost useless, since, in 1816, the following report was made to Government:

"I have seen *three*—certainly *two*—lunatics in one bed in the House of Industry (Dublin). I have seen, I think, not fewer than fifty or sixty persons in one room, of which, I believe, the majority were insane. I have seen in the same room a lunatic chained in a bed, the other half of which was occupied by a *sane* pauper." Such was, up to 1817, even in the metropolis of Ireland, the condition of the Irish lunatic.

With clear and recorded testimony such as the above, given by cool and dispassionate men, whose characters and integrity were beyond suspicion, and whose legitimate influence was very great, it became utterly impossible that such a state of things could longer exist without some remedy being applied. A Committee of the House of Commons was soon after appointed to devise the best means of meeting the evil. An able and comprehensive report (considering the time) was the result. This document, it is believed, was the production of a man, of whom his country may well be proud, (Lord Monteagle,) and whose labours for the improvement of his unfortunate country have been at all times steady and consistent. It was recommended to divide Ireland into districts,—to erect for each district a lunatic asylum, capable of containing 100 or 150 patients; the money requisite for the purpose to be advanced from the consolidated fund as a loan, repayable by the districts on certain conditions,—to place these new institutions exclusively under the control of the Lord-Lieutenant of Ireland. In the first instance, four; then six; and subsequently, ten districts were created, and ten asylums built: officers were appointed by the executive, and boards of superintendence, agreeably to the provisions of the act of parliament, were nominated by succeeding lords lieutenant. In fact, everything appeared to have been done to secure accommodation and care for such lunatics as obtained admission, *save one*,—that one omission was, that these asylums were considered rather as prisons than as hospitals. They were, in every instance, placed under civil governors or superintendents: one medical officer, a practising physician in the neighbouring town, was attached. He visited, according to the first regulations, twice a-week, unless when specially called for to accidents or sudden disease; in a word, he was often twenty miles away, attending his private avocations, when his immediate and pressing attendance might demand his professional duty at the asylum. The inspection of these institutions was placed under the prison department; in a word, the afflicted lunatic was looked on as a criminal, or an outcast, and treated as such, until recently; as in gaols, the medical attendant paid his occasional visit. Leg-locks, restraint-chains, bolts, and barred windows were supposed to do the rest.*

It was soon found that the accommodation provided in these new asylums, and which amounted to 1220, was too limited. The number swelled almost at once up to 2000, while the gaols, to which recent acts of parliament had facilitated admission, (inasmuch as gaols and lunatic asylums were bundled together by legislative and executive wisdom,) became so crowded by dangerous lunatics that all order and regularity seemed to be seriously impeded. In 1841, the lunatics confined in gaols amounted to 110; in 1843, they swelled to 214; and in 1849, notwithstanding the various causes which might be supposed to check the evil, the number again increased to 338, while the inmates of the district asylums were found to be 2603.

The following analysis of the lunatic poor of Ireland may more conclusively show their present condition than any lengthened remarks could do.

| No. 1. Paupers in District Asylums. | | No. 2. Paupers in Local Asylums. | | No. 3. Paupers in Gaols. | | No. 4. Workhouses. | | No. 5. Wandering Idiots or Lunatics. |
|---|---|---|---|---|---|---|---|---|
| Males | Fem. | Males | Fem. | Males | Fem. | Males | Fem. | About 6000 agreeably to Police rations. |
| 1348 | 1255 | 151 | 214 | 196 | 140 | 778 | 1162 | |
| 2603 | | 365 | | 338 | | 1940 | | |

Thus we find that although legislation *has* done something for the lunatic poor of Ireland, excepting those in columns 1, 2, 3, little if any provision exists for an overwhelming body of this unfortunate class.

It must be admitted, on all hands, that owing to the vigilance and care exercised by the present inspectors—Messrs. White and Nugent, the patients in column 1 are fully and steadily attended to, both in a moral and medical point of view, in such asylums as are provided with resident medical officers, and that, so far as it is possible in the remaining district asylums where such officers do not exist, the wants and comforts of the inmates are anxiously looked after. But from the following official statements in 1843, it is clear that in local pauper asylums the condition of the lunatic was dreadful in the extreme.

"Forty-nine lunatics have been confined in the local asylum of Kilkenny—twenty-five males and twenty-four females, with only one room in common to males and females for dinner. The day I visited

* The cost of restraints for one district asylum, some years ago, was officially returned for one year at £37 3*s*. 9*d*.

it there were twenty females at dinner in a small room—when they were done the males came in and dined."

At Wexford matters were as follow :—

"The state of the local asylum of Wexford is most disgraceful; one patient chained to a wall. He was naked, with a parcel of loose straw about him. He darted forward; and were it not that he was checked by a chain which went round his leg, and was fastened to the wall by a hook, he would have caught hold of me, and probably used violence. I went to another cell, and although not chained, the individual was almost as bad as the other. I went into another room, I looked around, I heard somebody moaning—on the top of a screen I saw two unfortunate lunatics stretched out, they were trying to warm themselves through the bars of a grating; the room was so dark that I could not see them at first."

Such is the recorded evidence of, perhaps, one of the mildest and most amiable public officers that ever existed—Dr. Francis White, inspector of prisons and lunatic asylums in 1843.

In reference to the condition of lunatics confined in the Irish workhouses, we would wish to drop a veil. The portrait is a terrible one. Their state cannot, perhaps, be paralleled in savage or civilized history.

We have now traced legislation and the actual condition of the Irish lunatic poor down to 1841; up to which period the history of her lunatic asylums is intimately interwoven; in fact, forms an essential part of the prison discipline of that country: a rapid change about that period commenced, and although checked in 1843-4, it has progressed in a most satisfactory manner since the latter year. Lord Mulgrave, as one of his last acts, nominated Dr. Stewart as manager or civil governor of Belfast. The local authorities, after much discussion with inspectors and others, deemed it desirable that his services as a medical man should be made available for the good of the public. The obsolete regulations prohibiting this were set aside. The change was most beneficial, and stood out in marked relief with other institutions painfully reported on by Sir David Barr, when officially visiting the public medical establishments of Ireland in 1841. Earl Fortescue nominated another medical gentleman, Dr. Flynn, to the Clonmel Asylum. The wedge was now effectually introduced. Upon a change of government, parties interested in keeping up the exclusion of medical men from their legitimate position, set themselves steadily, but, as the evidence given before parliament proves, stealthily, to work. The then Lord Chancellor of Ireland, Sir Edward Sugden, was enlisted against medical superintendence. His high name, his exalted character, and his pure, unostentatious benevolence, were all arrayed against this simple and humane provision, and in 1843 a code of regulations, under that great man's auspices, was run through the Privy Council of Ireland, which seemed to close every lunatic asylum, and for ever, against resident medical superintendence.

Monopoly and selfishness knew no bounds to their triumph—their annual local reports and parliamentary documents re-echoed the victory. Medical men, alas! for the profession—ay, even medical men, put forward their claims to public approbation, for being instrumental in excluding their own profession from its legitimate field of labour and of humanity. The victory was, however, of short duration: nobly did the English medical literature vindicate the profession. These regulations were also reviewed in France, Prussia, America—their absurd, contradictory, and vicious provisions, were exposed: the very authors of them at last felt ashamed of their wickedness; the transparent veil which covered the designs of their fabrications was torn asunder, and finally, the Report of the Lords' Committee, in 1843, on the State of the Lunatic Poor, laid the foundation for their permanent overthrow. Lord Ashley's Bill, in 1844, became law. None but medical men, henceforward, could, in England, be superintendents of her asylums. Sir James Graham carried to the working of the Bill the weight of government influence, and the anomalous spectacle was presented of a *legal* penalty being attached to any asylum in England having fifty patients and not having a medical officer residing and in charge of them, while in Ireland, under the same high functionary, as home secretary, no medical man, even if he happened to be a manager or civil governor of an Irish district lunatic asylum, and *as such* holding office immediately under Sir James Graham, could administer a dose of castor-oil to a patient within the walls of his asylum!

Human absurdity could go no further, and legislative interference came quietly to the rescue. In 1845 a new Lunatic Asylum Bill became the law; the prisons and asylums now parted company. Dr. White was appointed inspector, and in 1846 Dr. Nugent was added; and from this period a new and vigorous spirit has been evoked. In the first number of our Journal, we took the condition of the lunatic asylums in Ireland to task. Change after change has taken place; the old rules and regulations of 1843 have perished, in Carlow, Belfast, Limerick, Clonmel, and even in the cradle of their birth, Maryborough, by the appointment, as vacancies occurred, of resident physicians, or by the extension of their duties to such medical gentlemen as happened to have been managers. Much has thus been effected, but yet more remains behind.

Richmond Asylum, with 290 patients, under the very eye of the government, in Dublin, has no resident medical officer. Ballinasloe with 300 patients, has no resident medical officer. Cork, with its noble college, and its crowd of literary and scientific institutions, has no resident medical officer for its 420 patients. Neither has Derry, Waterford, nor Armagh. There are omissions in the every-day labours of a humane executive, which only need be pointed out to have a remedy at once applied.

What uniformity of action, of returns, or result, can government expect from the institutions under its immediate control, if so imperfect, so unwise, and we will even add, so barbarous a system is permitted to exist in this respect. Neither is it to be supposed for one moment, that with such vigorous intellects as those of Sir George Grey, Lord Clarendon, Sir William Somerville, and Sir Thomas Redington, things can remain as they are, if only the subject be fully and fairly considered in all its bearings by them. Let us, then, use all the energies of the press in England, and all the force which justice and humanity never fail to evoke in the minds of honest and able men, to carry out the full and fair provisions of our English system, so far as the remaining non-medical asylums of Ireland are concerned. All sections of society who wish well to the lunatic should take up this matter, and give their hearty and practical aid to the good cause. Our columns will be ever open to the advocates of this cause, and we trust ere long to record the most satisfactory results. In the meantime let us cheer on the Irish inspectors in their present arduous labours. They are deserving the full meed of public approbation and support, and we cheerfully and at once accord to them our warm thanks and gratitude on the part of our medical brethren.

Having so far touched on the government district lunatic asylums, it remains to say a few words on other establishments devoted, either wholly or in part, to the insane. Dean Swift, it is well known, left a fine and improving property for the care of idiots and insane persons: by the last available return it appears that there were sixty paying and eighty-two free inmates, but not a particle of information could be gleaned as to its financial arrangements; indeed, in a note to the report of the lunacy inspectors (page 30) for the year 1846-7, it is stated "that the expenditure for the year 1845 could not be ascertained." Admitting to the fullest extent that everything is well conducted; it is not seemly, in a Blue Book presented by command of her Majesty to parliament, that by the official organs of the government, so far as lunatic asylums are concerned, such a serious remark, calculated to create unpleasant feelings in the public mind, should be made. In the Island Bridge department of the Old House of Industry, Dublin, there appeared from the last return to have been about 329 idiots and lunatics confined, exclusively supported from a parliamentary grant, and which is now in gradual progress of extinction. It is time, in truth, that all lunatics should be placed in suitable institutions, as more in accordance with discipline and humanity; the extinction, therefore, of this establishment must be hailed as a still further step in advance.

The private asylums of Ireland are not numerous nor extensive; there are two in Cork, one in Armagh, eight in Dublin, one in Waterford, one in Limerick, and one in Maryborough; in public confidence they stand high. No complaint appears to have been ever recorded of injustice or cruelty; and the inspectors, in their annual Reports, speak in terms of warm approbation of the parties under whom they are conducted.

In conclusion, we have good reason to be satisfied with the progress of humane treatment towards the lunatics in Ireland; and when the five new district asylums now in progress of erection are completed; when all shall be placed under the charge of resident medical men of character and efficiency; and when the powers of the inspectors shall have been more fully recognised and consolidated; when, in fact, the executive will have organized an active system of co-operation between the as yet disjointed members of this department, Ireland may fairly claim a high position with regard to her public lunatic establishments among the other nations of the civilized world.

